# Metabolic oxidoreductases: central regulators of the epigenetic landscapes in stemness

**DOI:** 10.1038/s12276-026-01687-2

**Published:** 2026-04-13

**Authors:** Han-Teo Lee, Jae-Seok Roe, Hong-Duk Youn

**Affiliations:** 1 Stochastic Stemness Research Center, Department of Biomedical Sciences, Seoul National University College of Medicine, Seoul, Republic of Korea; 2 Ischemic/Hypoxic Disease Institute, Seoul National University College of Medicine, Seoul, Republic of Korea; 3 Department of Biochemistry, College of Life Science and Biotechnology, Yonsei University, Seoul, Republic of Korea

**Keywords:** Epigenetics, Embryonic germ cells

## Abstract

Cell metabolism has long been recognized as a fundamental process for energy production, biomolecule synthesis, and cell survival. During transitions between stem and somatic states, central carbon metabolism is rewired with concurrent changes in chromatin regulation and gene expression. Because a specific central carbon metabolic transition occurs simultaneously during the epigenetic transition from stem cells to somatic cells or vice versa, metabolism has an important role in determining the epigenetic landscape that influences cell fate. Oxidoreductases are key enzymes that catalyze oxidation–reduction reactions during central carbon metabolism. They also have important roles in determining the metabolic flux and epigenetic landscapes by supplying epigenetic metabolites, directly shaping chromatin structures through posttranslational modifications, and acting as scaffold proteins for epigenetic complexes to affect chromatin states. In this Review, we outline emerging mechanisms by which oxidoreductases couple metabolic flux to epigenetic landscapes that maintain or dissolve stemness.

## Energy flux shapes chromatin states in stem cells

Stem cells are special cells that can become any type of cell in the body. They have a unique ability called “stemness,” which allows them to regenerate tissues. Scientists have been studying stem cells for years to understand how they maintain this ability. A key part of this process involves something called the “epigenetic landscape,” which is how genes are turned on or off without changing the DNA sequence itself. Researchers have found that certain enzymes called oxidoreductases have a crucial role in this process. These enzymes help produce important molecules that modify the epigenetic landscape, influencing whether a stem cell remains a stem cell or becomes a different type of cell. The findings show that oxidoreductases not only support metabolism but also directly affect gene regulation by modifying chromatin, the structure that holds DNA.

This summary was initially drafted using artificial intelligence, then revised and fact-checked by the author.

## Introduction

Stem cells, including pluripotent stem cells and adult stem cells, exist in embryonic and postnatal tissues of mammals. These stem cells have an ability known as “stemness,” allowing them to generate all cells in the adult body or regenerate tissues within strictly limited potency^1^. Over the past 40 years, various stem cell types have been isolated from different tissues and cultured in vitro to understand the characteristics of stemness, such as naive versus primed pluripotency in embryonic stem cells (ESCs), epiblast stem cells (EpiSCs), induced pluripotent stem cells (iPSCs), and multipotency in adult stem cells^2^.

Stemness involves the precise spatiotemporal regulation of gene expression through coordinated control of gene clusters within the 3D chromatin structure. This dynamic chromatin environment, often referred to as the “epigenetic landscape,” shapes cell fate by enabling flexible gene regulatory programs. Specialized chromatin features in pluripotent stem cells include super-enhancers at identity genes^3^, bivalent histone domains at developmental gene promoters^4^, and global DNA hypomethylation^5^, which may endow stem cells with the ability to maintain stemness and influence cell fate decisions, extending beyond simple DNA sequences.

Epigenetic regulation occurs primarily through the combination of post-translational modifications of histones, chromatin remodelers, and specific nucleotides on DNA and RNA within chromatin regions. These modifications rely on enzymatic activities that use metabolites derived from central carbon metabolism^6^, indicating that metabolism is intertwined with the epigenetic regulation of chromatin states.

Cell metabolism has long been recognized as fundamental for energy production, biosynthesis, and cell survival. Notably, during transitions between stem and somatic states, central carbon metabolism is markedly rewired in parallel with changes in chromatin regulation and gene expression^7,8^. Because specific metabolic transitions coincide with epigenetic transitions between stem cells and differentiated cells, metabolism has an important role in determining the epigenetic landscape that influences cell fate^9,10^. Oxidoreductases are key enzymes in central carbon metabolism that catalyze oxidation–reduction reactions^11,12^. Beyond their metabolic roles, they help shape epigenetic states by supplying chromatin-modifying metabolites, directly modifying chromatin structures via post-translational modifications, and acting as scaffold proteins in epigenetic complexes^13,14^.

**Fig. 1:**
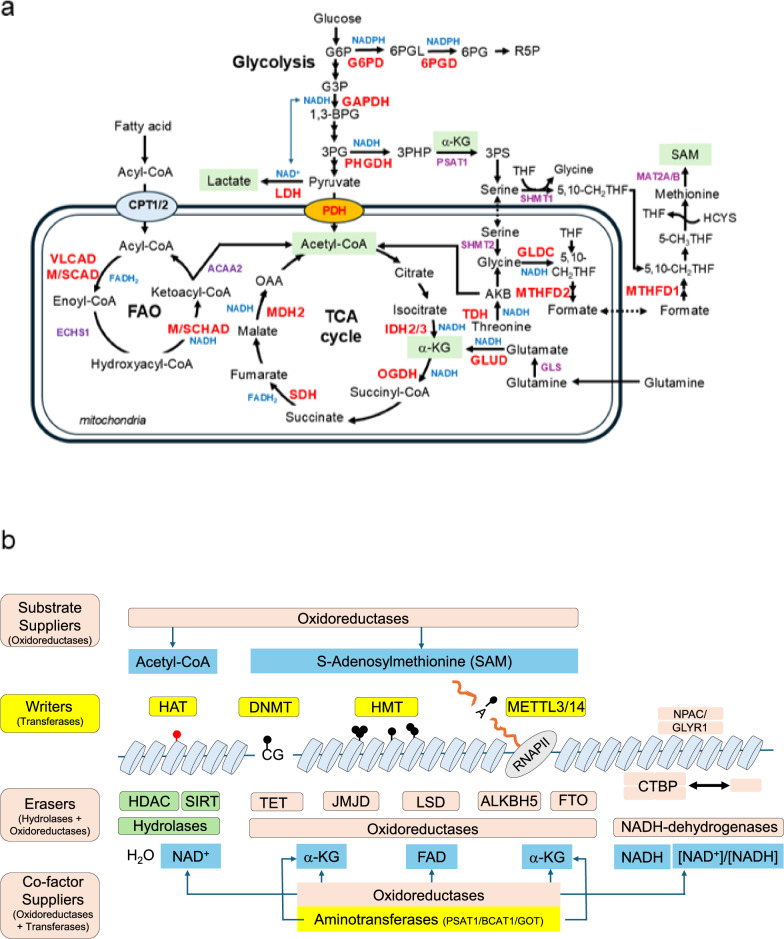
Oxidoreductases in epigenetic regulation. **a** Dehydrogenases generate critical epigenetic metabolites in the central carbon metabolic pathways. Pyruvate dehydrogenase (PDH) and FAO dehydrogenase supply acetyl-CoA; isocitrate dehydrogenase (IDH) and glutamate dehydrogenase (GLUD) directly produce KG; and lactate dehydrogenase (LDH) produces lactate. Threonine dehydrogenase is the rate-limiting enzyme for the production of acetyl-CoA and SAM in the mouse; phosphoglycerate dehydrogenase (PHGDH) is the rate-limiting enzyme for serine biosynthesis; and glycine dehydrogenase/decarboxylase (GLDC) produces 5,10-methylene-THF for SAM synthesis. Dehydrogenases are indicated in red. **b** Summary of oxidoreductases as key players in epigenetic regulation. Oxidoreductases are mainly responsible for supplying epigenetic metabolites. In epigenetic enzymes, all the writer proteins belong to the family of transferases. Hydrolases erase histone acetylation, whereas oxidoreductases erase histone/RNA/DNA methylation. AKB, 2-amino-3-ketobutyrate; ALKBH5, an Alkb homolog 5, RNA demethylase; CTBP, C-terminal binding proteins; DNMT, DNA methyltransferase; FAO, fatty acid oxidation; FTO, a fat mass and obesity-associated, RNA demethylase; G6P, glucose-6-phosphate; G6PD, glucose-6-phosphate dehydrogenase; HAT, histone acetyltransferase; HDAC, histone deacetylase; HMT, histone methyltransferase; LSD, lysine-specific demethylase; MCAD, medium-chain acyl-CoA dehydrogenase; MDH, malate dehydrogenase; METTL3/14, methyltransferase-like 3/14; NPAC/GLYR1, a glyoxylate reductase homolog 1; OAA, oxaloacetate; OGDH, α-ketoglutarate dehydrogenase; PSAT1, phosphoserine aminotransferase 1; SCAD, short-chain acyl-CoA dehydrogenase; SDH, succinate dehydrogenase; SIRT, sirtuin; TET, ten-eleven translocation methylcytosine dioxygenase; THF, tetrahydrofolate; VLCAD, very-long-chain acyl-CoA dehydrogenase; α-KG, α-ketoglutarate.

Conceptually, metabolic flux can be envisioned to generate an “energy contour” within the cell that in turn shapes an “epigenetic contour” on the chromatin. In other words, the peaks and valleys of metabolite and cofactor availability such as acetyl-CoA, NAD^+^/NADH, and α-ketoglutarate (α-KG) create a landscape of potential chemical modifications^13,15^ (Fig. 1). This energy contour is essentially imprinted onto chromatin as corresponding patterns of histone acetylation, DNA/histone methylation, and other epigenetic marks, thereby encoding the cell’s metabolic state into its gene-regulatory circuitry. This framework highlights how a cell’s energetic and redox state is intimately linked to its epigenetic status, setting a conceptual foundation for understanding mechanisms by which shifts in metabolism can drive changes in cell fate.

Oxidoreductases including dehydrogenases, oxygenases, oxidases, and peroxidases comprise a large family of enzymes responsible for redox reactions in central carbon metabolism^16^. Of these, oxygenases and dehydrogenases have important roles in epigenetic regulation. In central carbon metabolism, dehydrogenases primarily produce metabolites, such as acetyl-CoA, lactate, and α-KG, all of which are used as substrates/cofactors for epigenetic modifications (Fig. 1a and Table 1). Oxygenases use molecular oxygen for electron transfer from substrates. Oxygenases, such as JMJC domain (JMJD) histone demethylases, DNA 5-methylcytosine (5mC) dioxygenases (TET1/2/3), RNA 6-methyladenine dioxygenases (ALKBH5/FTO (a fat mass and obesity-associated protein)), and lysine-specific demethylase 1/2 (LSD1/2), regulate the methylation levels of histones and RNA/DNA, which are important for generating the epigenetic landscape (Fig. 1b). Furthermore, some dehydrogenases moonlight as structural components of chromatin-regulatory complexes. For instance, cytokine-specific nuclear factor/glyoxylate reductase 1 homolog (NPAC/GLYR1) functions as non-enzymatic scaffold proteins that recruit chromatin modifiers in ESC pluripotency networks (Fig. 1b).

**Table 1:** Oxidoreductases supply epigenetic metabolites for stemness.

Metabolic pathways	Enzymes	Cofactors/products	Epigenetic and metabolic features in stemness
Glycolysis	Glyceraldehyde-3-phosphate dehydrogenase (GAPDH)	NADH/1,3-bisphosphoglycerate	Produces NADH in payoff phase of glycolysis. In its knockout, quiescent HSCs sustain but proliferative HSCs are impaired
	Lactate dehydrogenase (LDH)	NADH/lactate	Reduces pyruvate to lactate for regeneration of NAD^+^ from NADH; lactate is used for histone lactylation
TCA cycle	Pyruvate dehydrogenase (PDH)	NADH/acetyl-CoA	Supplies glycolysis-derived acetyl-CoA; HIF1α-targeting pyruvate dehydrogenase kinases inhibit PDH in primed PSCs and quiescent adult stem cells
	Isocitrate dehydrogenases (IDH1/2/3)	NAD(P)H/α-KG	Supply α-KG for histone/DNA/RNA methylation at naive PSCs. In IDH1^R132^/IDH2^R172^ mutation in cancers, 2-hydroxyglutarate competitively distorts α-KG-dependent epigenetic regulation
	α-Ketoglutarate dehydrogenase (OGDH)	NADH/succinyl-CoA	Under hypoxia, SIAH2-mediated degradation of OGDH switches glutamate-derived α-KG to reduced lipid synthesis in cancer cells
	Succinate dehydrogenase (complex II) (SDH)	FADH_2_(Fe-S)/fumarate	SDHx mutations inhibit α-KG-dependent epigenetic regulation
	Malate dehydrogenases (MDH1/2)	NADH/oxaloacetate	MDH1-mediated malate–aspartate NADH shuttle for HSC self-renewal; MDH2-activated malate–aspartate shuttle reduces α-KG, resulting in ALKBH5-mediated epitranscriptome for maintaining GBM stemness
NADPH regeneration	Malic enzymes (ME1/2/3)	NADP^+^/pyruvate + CO_2_ + NADPH	NADPH acts as an endogenous inhibitor of HDAC3-NOR1; affects global histone acetylation in 3T3-L1 preadipocytes and their adipocyte differentiation
Pentose phosphate pathway	Glucose-6-phosphate dehydrogenase (G6PD)	NADP^+^/NADPH + 6-phosphogluconolactone	The rate-limiting enzyme for pentose phosphate; regenerates NADPH; affects histone acetylation in3T3-L1 preadipocytes
	6-Phosphogluconate dehydrogenase (6PGD)	NADP^+^/NADPH + ribulose 5-phosphate	The second enzyme for pentose phosphate regenerates NADPH
Aldehyde oxidation	Aldehyde dehydrogenase (ALDH)	NADH/carboxylic acid	Oxidize all-*trans*-retinal to all-*trans-*retinoic acid for stem cell differentiation; highly expressed in adult and cancer stem cells
Fatty acid oxidation (FAO)	Acyl-CoA dehydrogenases; very-long-chain acyl-CoA dehydrogenase (VLCAD), medium-chain acyl-CoA dehydrogenase (MCAD), and short-chain acyl-CoA dehydrogenase (SCAD)	FADH_2_/*trans*-Δ^2^-enoyl-CoA	Supply acetyl-CoA from fatty acids in naive PSCs and quiescent adult stem cells; oxidize the chain-length-specific acyl-CoA and chain-length-specific-hydroxyacyl-CoA; VLCAD is upregulated in AML; CPT1A or HADHA deficiency impairs HSC function as mice aged
	3-Hydroxyacyl-CoA dehydrogenases. Mitochondrial triple protein (HADHA/B); medium-chain/short-chain hydroxyacyl-CoA dehydrogenase (M/SHAD)	NADH/3-ketoacyl-CoA	
SAM synthesis	Threonine dehydrogenase (TDH)	NADH/2-amino-3-ketobutyrate	The rate-limiting enzyme for acetyl-CoA and glycine for SAM synthesis in mESCs; human TDH is pseudogene
	Glycine dehydrogenase/decarboxylase (GLDC)	NADH + THF/glycine + CO_2_ + NH_4_^+^ + 5,10-methylene-THF	Multisubunit complex (2P:27H;9T;1L); L subunit reduces NAD^+^ to NADH Transfer one-carbon from THF to 5,10-methylene-THF
Serine biosynthetic pathway	Phosphoglycerate dehydrogenase (PHGDH)	NADH/3-phosphoglycerate	The rate-limiting enzyme for serine biosynthesis; serine is used for one-carbon cycle
Glutaminolysis	Glutamate dehydrogenases (GLUD1/2)	NADH/α-KG	Replenish glutamate-derived α-KG into TCA cycle; supply α-KG for α-KG-dependent epigenetic regulation in adult and cancer stem cells
Kynurenine pathway	Indoleamine 2,3-dioxygenase 1 (IDO1)	O_2_/*N*-formylkynurenine	The rate-limiting enzyme for kynurenine metabolism; kynurenine-Ah receptor activates pluripotent genes and promotes glycolysis through the increase of [NAD^+^]/[NADH] ratio in primed hESCs; upregulated in many types of cancers
	Kynurenine 3-monooxygenase (KMO)	NADPH + O_2_/H_2_O + 3-hydroxykynurenine	Its inhibition decreases colorectal cancer stemness markers; upregulated in many types of cancers
	3-Hydroxyanthranilate dioxygenase (HAAO)	O_2_/quinolinic acid	A tumor suppressor; blocks 3-HA-mediated ferroptosis inhibition

*hESC* human embryonic stem cell, *HSC* hematopoietic stem cell *mESC* mouse embryonic stem cell, *PSC* pluripotent stem cell, *SAM* S-adenosylmethionine, *TCA* tricarboxylic acid, *THF* tetrahydrofolate, *α-KG* α-ketoglutarate, *3-HA* 3-hydroxyanthranilate, *GBM* glioblastoma, *AML* acute myeloid leukemia.

This Review aims to summarize the most recent information regarding oxidoreductases related to epigenetic regulation and to highlight their expanding role in shaping the epigenetic landscape for stemness.

## Enzymatic oxidoreductases

### Oxidoreductases produce metabolites for epigenetic regulation

#### NADH, NADPH, and FADH_2_

In the central carbon pathways, dehydrogenases reduce NAD^+^ (FAD) to NADH (FADH_2_) by transferring electrons/hydride from various molecules, such as carbohydrates, lipids, and amino acids (Fig. 1a and Table 1). For example, glycolytic glyceraldehyde-3-phosphate dehydrogenase (GAPDH) reduces NADH during the payoff phase of glycolysis. Interestingly, although GAPDH is essential for proliferative mouse hematopoietic stem cells (mHSCs), quiescent mHSCs can persist under GAPDH knockout conditions^17^. In the tricarboxylic acid (TCA) cycle, pyruvate dehydrogenase (PDH), isocitrate dehydrogenase (IDH), α-KG dehydrogenase, succinate dehydrogenase (SDH), and malate dehydrogenase (MDH) have important roles in conserving energy as reduced forms of NADH and FADH_2_ that connect to chromatin modification pathways. During fatty acid β-oxidation (FAO), acyl-CoA dehydrogenase and 3-hydroxyacyl-CoA dehydrogenase generate FADH_2_ and NADH, respectively^18^. Aldehyde dehydrogenase (ALDH) oxidizes aldehydes to carboxylic acids by reducing NAD^+^. They are highly expressed and considered stemness markers for detecting adult and cancer stem cells. Interestingly, ALDH is required for retinoid-driven hematopoietic differentiation in human and mouse HSCs, which suggests that high ALDH expression in stem cells may reflect a more differentiated state^19^.

NADH is primarily used for ATP generation for cell survival; however, it has a pivotal role in the changes occurring in the epigenetic landscape. Sirtuins (SIRT1–7), a family of NAD^+^-dependent histone deacetylases (HDAC), can remove acetyl groups from histones through the enzymatic cleavage of NAD^+^ to nicotinamide and 2ʹ-O-acetyl-ADP-ribose^20^. Sirtuins are considered energy or metabolic sensors because of their requirements for NAD^+^. The decreased ratio of [NAD^+^]/[NADH] during high glycolysis activity results in decreased SIRT1-mediated deacetylation of histones in muscle stem cells^21^.

Unlike NADH, NADPH is a cofactor for reductive biosynthesis. NADPH is produced by glucose 6-phosphate dehydrogenase and 6-phosphogluconate dehydrogenase in the pentose phosphate pathway, methylenetetrahydrofolate dehydrogenase, and malic enzyme. Interestingly, NADPH acts as an endogenous inhibitor of HDAC3 by interrupting the HDAC3–NCOR1 interaction. Knockdown of ME or glucose 6-phosphate dehydrogenase in 3T3-L1 preadipocytes results in decreased global histone acetylation and blocks differentiation to adipocytes, linking redox state to chromatin acetylation^22^.

##### Acetyl-CoA

Acetyl-CoA is the essential donor for histone acetylation. In human and mouse pluripotent stem cells, glycolysis is a major source of acetyl-CoA^23^. PDH in mitochondria converts pyruvate to acetyl-CoA, which enters the TCA cycle and can be exported as citrate for cytosolic acetyl-CoA production via ATP-citrate lyase (ACLY). In ESCs, high glycolytic flux sustains a pool of acetyl-CoA for robust histone acetylation. Inhibition of PDH by pyruvate dehydrogenase kinases (PDKs) diverts pyruvate to lactate instead of acetyl-CoA; accordingly, blocking PDKs with dichloroacetate in ESCs reroutes pyruvate into acetyl-CoA and increases histone acetylation. ESCs treated with glycolysis inhibitors or PDH blockers exhibit reduced histone acetylation and spontaneously differentiate, underscoring the importance of glycolysis-derived acetyl-CoA in maintaining an open chromatin, hyperacetylated state that supports pluripotency^24,25^.

**Fig. 2:**
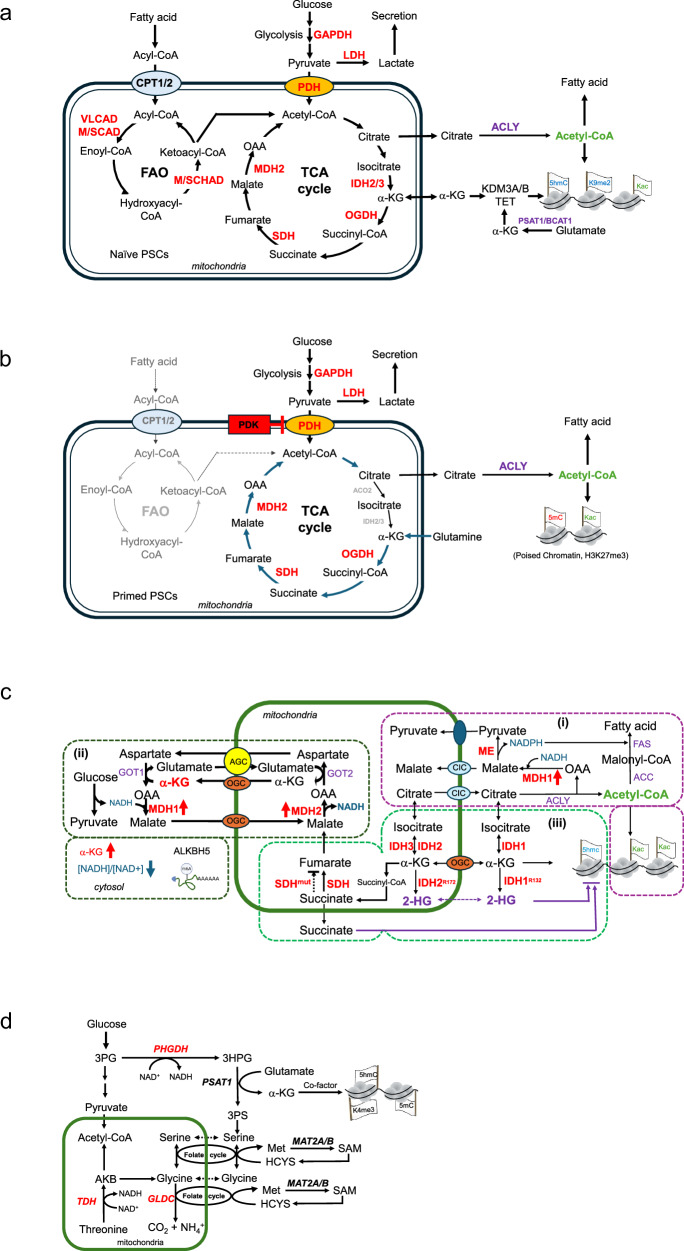
The roles of oxidoreductases in the signature of metabolic pathways for stemness. **a** Naive pluripotent stem cells (PSCs) can produce acetyl-CoA from glycolysis and fatty acid oxidation (FAO). This acetyl-CoA is further utilized to produce NADH and FADH_2_ through oxidative phosphorylation. Surplus citrate is transported into the cytosol and then cleaved back to acetyl-CoA, which is utilized for histone acetylation in PSCs. **b** Primed PSCs primarily rely on glycolysis, whereas FAO is not utilized. Inhibition of pyruvate dehydrogenase (PDH) by pyruvate dehydrogenase kinase (PDK) leads to the conversion of pyruvate to lactate instead of acetyl-CoA. In this state, glutamate is converted to α-ketoglutarate (α-KG), which replenishes the tricarboxylic acid (TCA) cycle in primed PSCs. The acetyl-CoA produced in this metabolic state serves as a substrate source for histone acetylation, contributing to the establishment of poised chromatin. **c** Metabolite shuttle system in stemness. (i) Citrate is transported into the cytosol by the citrate (tricarboxylate) carrier (CIC). Citrate is cleaved into oxaloacetate (OAA) and acetyl-CoA. Acetyl-CoA is utilized for fatty acid synthesis and histone hyperacetylation in PSCs. Malate dehydrogenase 1 (MDH1) may reduce OAA to malate, which is returned to the mitochondria, or the malic enzyme (ME) further oxidizes malate to pyruvate, which enters the mitochondria. (ii) In hematopoietic stem cells (HSCs), MDH1 activates the malate–aspartate activated by MDH1 resulting in a reduced cytosolic [NADH]/[NAD^+^] ratio, which sustains both glycolysis and oxidative phosphorylation for HSC’s self-renewal. In glioblastomas, the MDH2-activated malate–aspartate shuttle leads to the accumulation of cytosolic α-KG, which affects the ALKBH5-mediated epitranscriptome. (iii) α-KG is transported to the cytosol. Heterozygous mutations in cytosolic IDH1^R132^ or mitochondrial IDH2^R172^ result in 2-HG production, which suppresses α-KG dependent dioxygenases and ultimately affects the chromatin states in cancer stem cells. SDHx mutations also accumulate succinate, which inhibits α-KG-dependent dioxygenases in cancer stem cells. **d** Threonine dehydrogenase (TDH) is the rate-limiting enzyme that oxidizes threonine to 2-amino-3-ketobutyrate (AKB), subsequently providing acetyl-CoA and glycine for SAM synthesis. Phosphoglycerate dehydrogenase (PHGDH) is the rate-limiting enzyme in serine biosynthesis, with endogenous serine providing one-carbon units for SAM synthesis. ACLY, ATP-citrate lyase; GAPDH, glyceraldehyde-3-phosphate dehydrogenase; GLDC, glycine dehydrogenase/decarboxylase; IGH, isocitrate dehydrogenase; LDH, lactate dehydrogenase; MCAD, medium-chain acyl-CoA dehydrogenase; OGDH, α-ketoglutarate dehydrogenase; PSAT1, phosphoserine aminotransferase 1; SCAD, short-chain acyl-CoA dehydrogenase; SDH, succinate dehydrogenase; TET, ten-eleven translocation methylcytosine dioxygenase; VLCAD, very-long-chain acyl-CoA dehydrogenase; 2-HG, 2-hydroxyglutarate.

Besides glucose, FAO can contribute substantially to the acetyl-CoA pool in certain contexts. In differentiated hepatocytes, FAO provides up to 90% of acetyl-CoA for histone acetylation, promoting lipid metabolic gene expression^26,27^. However, conventional (primed) human iPSCs rely minimally on FAO for energy or acetyl-CoA production^24^, and chemically modulating FAO in primed hPSCs has little effect on pluripotency markers^23^. By contrast, naive PSCs (which have a more preimplantation-like, metabolically oxidative state) do utilize FAO as an energy source^28,29^. This suggests that FAO-derived acetyl-CoA can support the epigenetic landscape of naive pluripotency (Fig. 2a). Interestingly, when mESCs are induced into a “diapause-like” dormancy (either by Myc depletion or by mTOR inhibition)^30,31^, they increasingly rely on FAO for ATP and acetyl-CoA production^32,33^. In these dormant pluripotent cells, global histone acetylation (for example, H4K16ac) is reduced; however, FOXO1-driven CPT1A expression sustains FAO flux and acetyl-CoA levels to maintain viability. Conversely, experimentally forcing a diapause-like state by deleting the histone acetyltransferase (HAT) males absent on the first (MOF, KAT8/MYST1) in naive mESCs diminishes FAO gene expression (through loss of H4K16ac at those loci) and triggers a quiescent pluripotent state akin to diapause, which can be partially rescued by providing fatty acid substrates^28^. Thus, depending on the pluripotent state and environmental conditions, cells can flexibly use glycolysis or FAO for acetyl-CoA to support histone acetylation.

Most adult stem cells in vivo are quiescent and predominantly utilize FAO and oxidative phosphorylation (OXPHOS), rather than glycolysis, to meet their energy needs. Quiescent HSCs in both human and mouse and neural stem cells (NSCs) in mouse, for example, depend on the FOXO1–CPT1A axis and FAO to maintain self-renewal capacity in low-nutrient or hypoxic niches^34,35^. It remains to be fully explored whether FAO-derived acetyl-CoA in these adult stem cells substantially contributes to histone acetylation or whether other pathways compensate under quiescence. Some evidence suggests that quiescent mHSCs favor anaerobic glycolysis over OXPHOS in hypoxia^36,37^, and that cytosolic redox shuttles (for example, malate-aspartate shuttle via MDH1/MDH2) help balance NADH to sustain both glycolysis and OXPHOS in mouse HSCs during self-renewal and in human glioblastoma stem cells^38,39^ (Fig. 2c). In cancer stem cell models, FAO has been implicated in maintaining stemness and driving specific differentiation programs^39^, indicating that acetyl-CoA from FAO can influence the epigenome during development and in disease contexts.

#### Lactate

At high glycolytic rates, lactate dehydrogenases oxidize pyruvate to lactate to regenerate NAD^+^ from NADH. Long considered a waste product, lactate can modulate the stem cell environment and epigenome. Highly glycolytic stem cells (for example, primed PSCs) export lactate to acidify their microenvironment^40^. Lactate itself has been reported to promote stem cell properties in some contexts — for example, by acting as an energy source in cancer stem cells or by inducing a pseudohypoxic state that maintains pluripotency gene expression^41^. Lactate may also indirectly support stemness by increasing extracellular acidity and inhibiting differentiation signals. Furthermore, lactate can be metabolically “coupled” to histone modifications: excess lactate inside the cell can be converted to lactyl-CoA, which non-enzymatically acylates histone lysines to form histone lactylation marks (such as H3K18-lactyl)^40^. Histone lactylation has been associated with activation of gene programs including those involved in glycolysis, potentially creating a positive feedback loop sustaining a high glycolysis state in stem cells^42^.

By contrast, opposing evidence suggests that lactate can drive differentiation. In mouse ESC cultures, adding excess lactate or forcing reliance on glycolysis led to spontaneous differentiation and specifically promoted differentiation toward extra-embryonic endoderm lineages^43,44^. In human ESCs undergoing directed differentiation, intracellular lactate levels rise during neuroectodermal differentiation, coincident with a surge in H3K18 lactylation marks at genes associated with development^45^. These findings imply that the effect of lactate on stemness is context-dependent: lactate and histone lactylation can either support a stem-like state or push differentiation, depending on the cell type and metabolic milieu. Intriguingly, histone acetylation and lactylation both mark active chromatin and increase transcription; however, they originate from different metabolic conditions (acetyl-CoA can derive from both oxidative metabolism and glycolysis, whereas lactate accrues primarily under high glycolysis/low PDH activity). It remains an open question how cells interpret these two marks. The interplay between acetylation and lactylation might fine-tune gene expression based on metabolic state. For instance, a high glycolytic state (high lactate) might shift the balance from acetylation to lactylation on certain histones, potentially altering promoter and enhancer activity in ways that we are just beginning to understand^42^. Further studies are needed to delineate the precise roles of histone lactylation versus acetylation in maintaining stemness or triggering differentiation.

#### α-Ketoglutarate

α-KG is a central TCA metabolite and an essential cofactor for α-KG-dependent dioxygenases, including the TET DNA demethylases and JMJC histone demethylases. Sufficient levels of α-KG favor these demethylation reactions, whereas accumulation of certain metabolites such as 2-hydroxyglutarate, succinate, or fumarate (which can occur owing to IDH mutations or SDH/fumarase deficiencies) competitively inhibits α-KG-dependent enzymes^12,46,47^. Primed hPSCs cannot use pyruvate efficiently because of the poor expression of ACO2 and IDH2/3 in the TCA cycle and PDK-mediated inactivation of PDH. Thus, primed hPSCs rely on glycolysis and glutaminolysis, in which glutamine is subsequently catalyzed to α-KG, replenishing the TCA cycle^48^ (Fig. 2b). However, naive hPSCs showed reduced independence for glutamine anaplerosis^49^. Similar to primed hPSCs, mESCs in the serum/leukemia inhibitory factor (LIF) culture use glutamine and glucose to maintain high levels of α-KG, albeit naive mESCs in the 2i/LIF culture maintain pluripotency in the absence of exogenous glutamine. Consequently, mESCs in the presence of glutamine exhibit an elevated ratio of α-KG to succinate, which promotes the demethylation of histones and DNA, thus maintaining pluripotency^13^. In mESCs, aminotransferases, such as branched-chain aminotransferase 1 and phosphoserine aminotransferase 1, are the predominant enzymes responsible for the conversion of glutamate to α-KG, which is essential for maintaining the epigenetic landscape in mESCs^50,51^ (Fig. 2a,d).

In addition to aminotransferases, glutamate dehydrogenase (GLUD) may also contribute to α-KG production, although its role in pluripotent stem cells remains unclear. In human hematopoietic stem cells, GLUD converts glutamate to α-KG, which feeds into the TCA cycle^52^. GLUD1 catalyzes this reaction in many cancer types, and its expression compensates for α-KG deficiency in IDH1-mutant gliomas^53,54^. Interestingly, GLUD1/2 inhibition impairs redox homeostasis and proliferation in these cells. In adult stem cells, GLUD1-derived α-KG has been shown to promote demethylation of repressive histone marks (for example, H3K9me3 and H3K27me3), enhancing regeneration and differentiation. In neurons, mitochondrial GLUD can even translocate to the nucleus to support TET3-mediated epigenetic remodeling during neural plasticity^55^. These findings suggest that GLUD-mediated α-KG supports histone, DNA, and RNA demethylation in both cancer and somatic stem cell contexts.

**Fig. 3:**
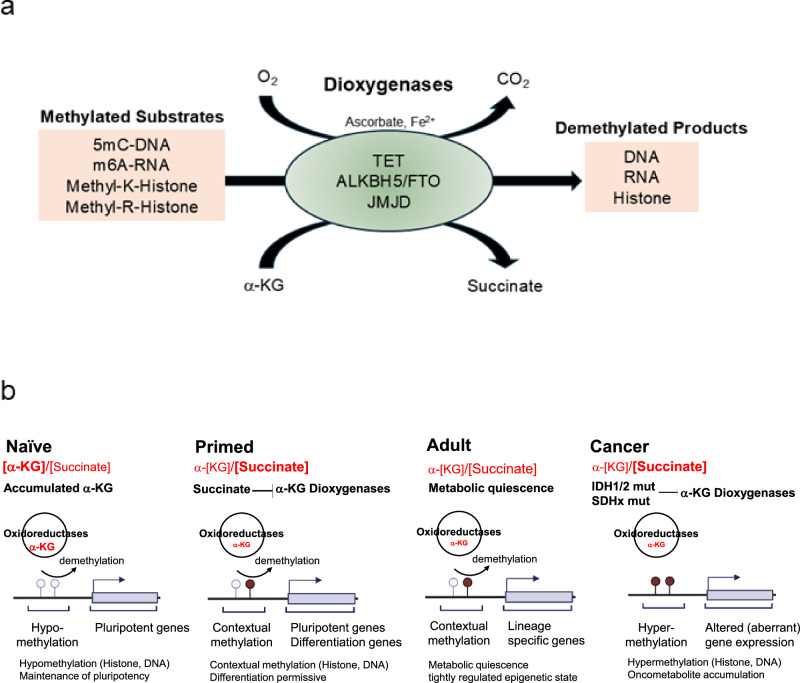
α-KG-dependent dioxygenases in stemness. **a** Schematic model of α-ketoglutarate (α-KG)-dependent dioxygenases. α-KG-dependent dioxygenases convert methylated substrates to demethylated products by hydroxylation in an O_2_/α-KG-dependent manner. Ascorbate reduces oxidized Fe^3+^ to reduced Fe^2+^. **b** The ratio of α-KG to succinate affects gene expression related to stemness. The increased α-KG to succinate ratio in naive pluripotent stem cells activates DNA and histone demethylation and induces the expression of pluripotency genes. In primed pluripotent stem cells, a decreased α-KG to succinate ratio inhibits α-KG dioxygenases, resulting in gene expression changes based on the cellular context. In dormant adult stem cells, metabolism is maintained to keep cells in a quiescent state. In IDH1^R132^ and IDH2^R172^ mutations, the oncometabolite 2-hydroxyglutarate inhibits α-KG dioxygenases. Similarly, in SDHx mutations, the accumulation of succinate or fumarate also inhibits α-KG dioxygenases, which results in aberrant gene expression. IDH, isocitrate dehydrogenase; LSD, lysine-specific demethylase; m6A, N6-methyladenine; SDH, succinate dehydrogenase; TET, ten-eleven translocation methylcytosine dioxygenase; 5mC, 5-methylcytosine.

By contrast, increased α-KG accelerates the initial differentiation by promoting demethylation reactions that activate lineage-specific genes, but silence pluripotency genes in primed hPSCs and mEpiSCs. These opposite effects between naive and primed pluripotency result from the context-specific role of α-KG^56^. Importantly, it is not just the absolute α-KG concentration but the “ratio” of α-KG to succinate that matters for enzyme activity (Fig. 3b). Naive PSCs tend to have a high [α-KG]/[succinate] ratio because active PDH and FAO supply α-KG (through enhanced TCA flux), whereas succinate is efficiently oxidized via SDH. In primed PSCs, PDH is inhibited and IDH2/3 activity reduced, leading to lower α-KG production; these cells rely on glutaminolysis for α-KG, yet also accumulate more succinate (through a truncated TCA and other anaplerotic fluxes), resulting in a lower [α-KG]/[succinate] ratio. The lower ratio in primed cells could partially suppress TET/JMJ demethylation, helping maintain the higher DNA/histone methylation characteristic of primed pluripotency, whereas the higher ratio in naive cells permits more active demethylation and a hypomethylated state. In adult quiescent stem cells, which are highly FAO-dependent, α-KG levels and the α-KG/succinate ratio are expected to be low, potentially restraining demethylase activity and locking cells in a more methylated, quiescent chromatin state.

#### *S*-adenosylmethionine

Another metabolite intimately linked to epigenetics is *S*-adenosylmethionine (SAM), the universal methyl donor for DNA, RNA, and histone methyltransferases. One-carbon (1C) metabolic flux from amino acids such as threonine and serine contributes to SAM levels in mouse ESCs. Threonine is uniquely required by mouse ESCs to sustain proper SAM levels: threonine dehydrogenase (TDH) oxidizes threonine to 2-amino-3-ketobutyrate, which is converted into acetyl-CoA and glycine, feeding into the folate cycle and ultimately SAM synthesis^57,58^ (Fig. 2d). In mESCs, threonine deprivation or TDH inhibition causes SAM depletion, loss of H3K4me3, and differentiation. Notably, the glycine cleavage system (GLDC enzyme complex) in mitochondria is a TDH-coupled oxidoreductase that channels glycine-derived one-carbon units into 5,10-methylenetetrahydrofolate (5,10-CH_2_-THF), supporting methionine cycling and SAM production. Human PSCs, however, lack functional TDH (TDH is a pseudogene in humans) and instead rely on exogenous methionine to produce SAM via MAT2A/B. Methionine withdrawal in hPSCs rapidly reduces cellular SAM and H3K4me3, leading to differentiation and apoptosis^59^. Interestingly, naive hESCs show high expression of nicotinamide *N*-methyltransferase (NNMT), which consumes SAM to methylate nicotinamide, thereby lowering SAM and enforcing a hypomethylated H3K27me3-low state associated with naive pluripotency^29^. This illustrates how metabolic tuning of SAM levels (for example, through NNMT) can modulate epigenetic methylation marks during different pluripotent states.

Serine-derived one-carbon units also contribute to the SAM pool. The enzyme phosphoglycerate dehydrogenase diverts glycolytic 3-phosphoglycerate into serine synthesis, and serine can be converted by serine hydroxymethyltransferases (SHMT1/2) to glycine while generating 5,10-CH_2_-THF^60^.

#### Kynurenine

Tryptophan metabolism is important for self-renewal and the pluripotency of stem cells. Indoleamine 2,3-dioxygenase 1 (IDO1) is the rate-limiting oxidoreductase that converts tryptophan to *N*-formylkynurenine, which is then converted to kynurenine by formamidase. Kynurenine is highly abundant in primed PSCs compared with naive PSCs^29^. It maintains the undifferentiated state in primed hPSCs by acting as an agonist for the aryl hydrocarbon receptor (AhR), and promoting pluripotent genes and IDO1 as a positive feedback mechanism^61^. Moreover, kynurenine promotes glycolysis by increasing the [NAD^+^]/[NADH] ratio in primed hESCs^62^. By contrast, AhR is minimally expressed in hPSCs; thus, kynurenine maintains proliferation and pluripotency independent of AhR signaling^63^. Downstream enzymes in the kynurenine pathway also have roles. Kynurenine 3-monooxygenase (KMO) oxidizes kynurenine to 3-hydroxykynurenine using NADPH and molecular oxygen. Inhibiting KMO results in decreased stemness markers in colorectal cancers, which are upregulated in many cancer types^64^. 3-Hydroxyanthranilate dioxygenase (HAAO) oxidizes 3-hydroxyanthranilate (3-HA) to 2-amino-3-carboxymuconic semialdehyde, which is non-enzymatically cyclized to quinolinic acid. HAAO has an important role in supplying a precursor for de novo NAD biosynthesis and acts as a tumor suppressor to block 3-HA-mediated ferroptosis inhibition^65,66^.

#### Brief summary

This section underscores a central principle of metabolic–epigenetic coupling, emphasizing that metabolite availability is itself a regulatory signal. Oxidoreductases do more than maintain metabolic flux. They actively set the biochemical boundaries within which chromatin-modifying enzymes operate. By generating key cofactors such as acetyl-CoA, NAD⁺/NADH, and α-KG, they define the “metabolic potential” of a cell to write, erase, or maintain epigenetic marks. In this view, oxidoreductases shape the energy contour that ultimately constrains or permits specific epigenetic contours. This concept frames metabolism as an upstream designer of chromatin states, linking cellular physiology directly to gene-regulatory outcomes.

#### Oxidoreductases directly regulate the epigenetic states of chromatin

Two types of oxidoreductases contribute to changes in the epigenetic states of chromatin: (1) the Fe(II)/α-KG-dependent dioxygenases and (2) the FAD-dependent amine oxidases.

**Fig. 4:**
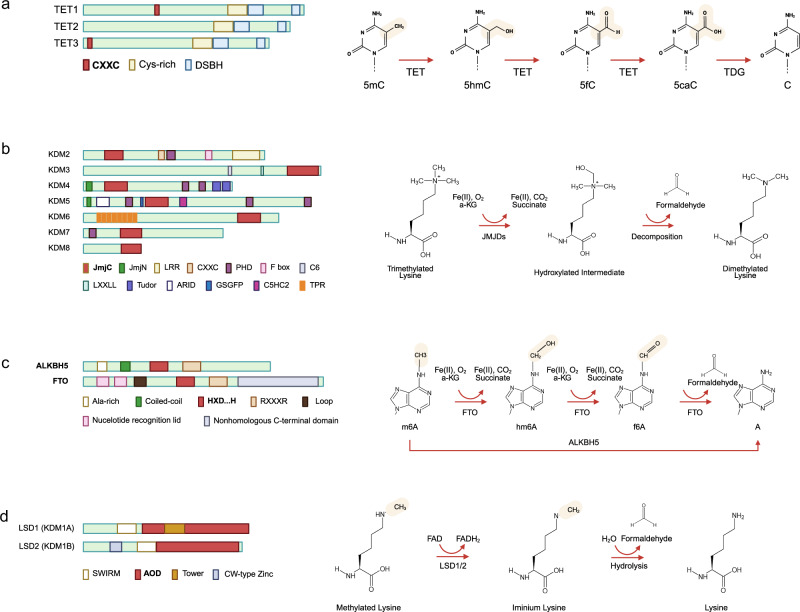
Primary structures of epigenetic oxidoreductases and their reaction mechanisms. **a** TET iteratively oxidizes 5-methylcytosine (5mC) to form 5-hydroxymethylcytosine (5hmC), 5-formylcytosine (5-fC), and 5-carboxycytosine (5caC) using α-ketoglutarate. 5fC and 5caC are excised by thymine-DNA glycosylase (TDG). **b** JMJDs demethylate the dimethyl-lysine and trimethyl-lysine to monomethylated and demethylated lysine by hydroxylation using α-ketoglutarate as a cofactor, followed by decomposition. Each type of JMJD isoform has substrate specificity summarized in Table 2. **c** FTO successively demethylates N6-methyladenine (m6A) to N6-hydroxymethyladenine (hm6A), N6-formyladenine (f6A), and then adenine (A). ALKBH5 directly demethylates m6A to A. **d** LSD1 demethylates dimethyl-lysine and monomethyl-lysine to mono-lysine and naked lysine in an FAD-dependent manner. Their substrate specificity is described in Table 2. LSD1/2 is limited to the demethylation of monomethyl-lysine and dimethyl-lysine residues. DSBH, double-stranded β-helix; FTO, a fat mass and obesity-associated protein; TET, ten-eleven translocation methylcytosine dioxygenase.

The former includes the TET family of DNA demethylases, the JMJ family of histone demethylases, and the RNA demethylases ALKBH5 and FTO. They catalyze the oxidation of methylated substrates to hydroxylated products, using molecular oxygen. Simultaneously, α-KG is converted into succinate and carbon dioxide, which require reduced iron (Fe^2+^) for substrate oxidation. Consequently, the oxidized iron (Fe^3+^) is reduced by ascorbate (Fig. 3a). The latter group comprises LSD1 and LSD2, which use FAD to oxidatively demethylate histone lysines, producing imines that are hydrolyzed to formaldehyde (Fig. 4d). Notably, each stem cell type or pluripotency state exhibits a distinct metabolic profile that can modulate the activity of these oxidoreductases (Fig. 3b). Although this concept appears to be generally applicable, the specific chromatin signatures will vary depending on the context of the stem cells and the enzyme type and expression. A comprehensive understanding will require further studies in the future. In this Review, we aimed to describe the molecular properties of these enzymes and their roles in maintaining stemness.

#### DNA 5-methylcytosine dioxygenases: TET1/2/3

##### Molecular properties

A family of ten-eleven translocation proteins was initially discovered in two patients with leukemia as a translocation partner with mixed lineage leukemia. They specifically involved the chromosomal translocation t(10;11)(q22;q23), which resulted in its designation as a ten-eleven translocation^67,68^. The TET isoforms (TET1/TET2/TET3) iteratively oxidize 5mC to form 5-hydroxymethylcytosine (5hmC), 5-formylcytosine (5fC), and 5-carboxycytosine (5caC), using α-KG as a cofactor^69,70^ (Fig. 4a). Subsequently, 5fC and 5caC are recognized and excised by thymine-DNA glycosylase, resulting in the restoration of unmethylated cytosine^71,72^.

The TET proteins are structurally characterized by a CXXC-type zinc finger domain and a catalytic Fe(II)-dependent and α-KG-dependent dioxygenase domain, which includes a cysteine-rich domain (Fig. 4a). The CXXC domain has an important role in recognizing DNA methylation status by specifically binding to unmethylated CpG sequences^73^. Unmethylated CpG sites are typically abundant in active regulatory elements, such as promoters and enhancers. This physically protects significant genes from CpG methylation by DNA methyltransferases (DNMTs) and ensures that DNA methylation does not excessively progress before TET proteins initiate DNA demethylation at these sites^74^.

Unlike TET1 and TET3, TET2 lacks a CXXC domain, which is instead encoded by a separate gene, IDAX (CXXC4). IDAX binds unmethylated CpG sites and interacts with the catalytic domain of TET2, guiding its localization and regulating its protein stability^75^. Through this interaction, IDAX compensates for the absence of CXXC and helps maintain proper DNA demethylation activity.

The catalytic domain of the TET proteins contains two key subdomains: the double-stranded β-helix (DSBH) domain and the Cys-rich domain.

The DSBH domain adopts a β-sandwich structure consisting of two layers of antiparallel β-strands that form a helical arrangement. Within this domain, Fe(II) ion is coordinated by a conserved triad of amino acid residues, usually two histidines and one aspartate or glutamate. The catalytic site is arranged to position 5mC from the DNA in close proximity to the Fe(II) and α-KG, which allows for efficient oxygen transfer during the oxidation process. Although the DSBH domain itself does not directly bind to DNA, it interacts with DNA through the broader catalytic domain, which includes the Cys-rich insertion.

The Cys-rich domain contributes to the structural stability of the catalytic domain. It acts as a specificity-determining element, enabling TET proteins to more effectively recognize target DNA sequences. In addition, the Cys-rich domain participates in interactions with other proteins, thereby enhancing enzyme activity and contributing to the precise regulation of DNA demethylation.

Taken together, the DSBH domain has an essential role in catalysis, whereas the Cys-rich domain supports structural integrity and specificity, maximizing the efficiency and precision of DNA demethylation and epigenetic regulation in cells.

##### Epigenetic role of TET1/2/3 in stemness

During early embryogenesis, DNA methylation changes dynamically. After fertilization, the paternal genome rapidly loses its methylcytosine marks, even before DNA replication, whereas the maternal genome undergoes passive demethylation during the early cell cycle before blastulation. Proper regulation of DNA methylation and demethylation is necessary for proper development^76^.

TET1 and TET2 are predominantly expressed in the inner cell mass of preimplantation mouse embryos and maintain 5hmC levels^77^. High TET1 and TET2 expressions contribute to elevated 5hmC levels in mESCs and hiPSCs, which are associated with pluripotency^77–79^; however, they play opposing roles in regulating the pluripotent states in ESCs, with TET1 supporting naive pluripotency and TET2 promoting primed pluripotency. This distinction arises from their differential interactions with chromatin regulators or target gene promoters. Zfp281 serves as a regulator by interacting with TET1 to suppress TET2 expression, thereby maintaining primed states, while inhibiting the transition to naive states^80^. This coordination reveals a molecular circuitry that balances the acquisition and maintenance of distinct pluripotent states.

In mESCs, TET1 occupies both transcriptionally active genes and Polycomb-repressed developmental regulators^81^. TET1-mediated promoter hypomethylation is essential for maintaining the expression of genes related to pluripotency and self-renewal in ESCs. Conversely, TET1 facilitates PRC2 recruitment to CpG-rich promoters of developmental regulators. These complementary functions highlight the importance of TET1 in maintaining the pluripotent state and regulating cell fate decisions.

Despite its significance, TET1 depletion does not abolish self-renewal under standard conditions, but TET1-null mESCs show skewed differentiation biases and aberrant methylation at specific loci, indicating that TET1 is required for proper lineage specification rather than core pluripotency maintenance^77^. Indeed, TET1 knockout mESCs remain pluripotent but exhibit differentiation defects, such as excessive trophoblast differentiation at the expense of embryo-proper lineages.

TET2 appears less critical in mESCs but very important in hematopoietic stem/progenitor cells. TET2 is frequently mutated in hematological malignancies, and Tet2 loss in mice leads to increased HSC self-renewal and eventual myeloid transformation^82,83^. In mESCs, TET2 largely overlaps functionally with TET1; only combined Tet1/Tet2 double knockout causes pronounced 5mC accumulation and developmental failure, although some double-knockout mESCs can still contribute to chimeras^84,85^. Triple knockout of Tet1/2/3 is embryonic lethal and underscores that some minimal DNA demethylation activity is essential for normal development.

TET3 expression varies depending on the stem cell type and its developmental stage. TET3 is highly expressed in oocytes, where it facilitates paternal genome demethylation^86^; however, it is barely detectable in stem cells, but increases during neuroectoderm differentiation. Catalytic mutant and knockout TET3 ESCs indicate that its activity is important for neural gene activation, whereas its non-catalytic functions are involved in suppressing mesodermal programs^87^. This highlights its role in early neural lineage commitment, distinguishing it from TET1 and TET2. TET1/2 double-knockout ESCs result in 5hmC depletion, including at imprinted loci, which causes developmental defects. Nonetheless, only a fraction of DKO mice are compatible with postnatal development and show no overt morphological defects, indicating that TET3 partially compensates for the loss of TET1 and TET2 (ref. ^84^).

#### JMJD histone demethylases

##### Molecular properties

JMJD histone demethylases generally demethylate dimethyl-lysine and trimethyl-lysine to monomethyl-lysine and dimethyl-lysine by the hydroxylation of α-KG and iron (Fe²⁺) as essential cofactors, followed by decomposition (Fig. 4b). Each JMJD isoform has its substrate specificity (Table 2). These enzymes are important for histone lysine demethylation and have an important role in epigenetic regulation, cellular differentiation, development, and disease progression^88,89^. Members of the JMJD family exhibit substrate-specific activity and target distinct histone lysine residues and methylation states through sequence homology and unique structural features.

**Table 2:** Oxidoreductases as epigenetic enzymes.

Enzymes	Catalytic activities	Epigenetic roles in stemness
TET1/2/3	Subsequent hydroxylation of 5mC to 5hmC, 5fC, 5caC	Maintain an open chromatin state by promoting DNA demethylation at pluripotency gene promoters; regulate proper lineage commitment during differentiation
JHDM1A/B (KDM2A/B)	Demethylation of H3K36me_1/2_; H3K4me_3_; H3K79me_2/3_ (KDM2B)	Suppress inappropriate transcription by maintaining repression at non-coding regions and maintaining genome stability in stem cells
JMJD1A/B/C (KDM3A/B/C)	Demethylation of H3K9me_1/2_	Activate transcription of stemness and developmental genes by removing repressive H3K9 methylation marks
JMJD2A/B/C/D/E/F (KDM4A/B/C/D/E/F)	Demethylation of H3K9me_2/3_; H3K36me_2/3_ (not JMJD2D/E)	Increase chromatin accessibility and support transcriptional activation of pluripotency-associated genes; enhance reprogramming efficiency
JARID1A/B/C/D (KDM5A/B/C/D)	Demethylation of H3K4me_2/3_	Maintain bivalent chromatin states at developmental loci, allowing flexible activation or repression during stem cell fate transitions
UTX/JMJD3/UTY (KDM6A/B/C)	Demethylation of H3K27me_2/3_	Facilitate activation of developmental genes by removing Polycomb-mediated repressive marks, thus driving exit from pluripotency and commitment to differentiation
KIAA1718/PHF8/PHF2 (KDM7A/B/C)	Demethylation of H3K9me_1/2;_ H3K27me_1/2_, H4K20me_1_ (KDM7A, PHF8)	Promote lineage-specific gene expression programs by erasing repressive histone marks and enhancing transcription initiation
JMJD6	Demethylation of H3R2me_1/2_, H4R3me_1/2_; C5 hydroxylation of lysine	Enhance self-renewal capacity by regulating transcription elongation and metabolism in ESCs and HSCs; support somatic cell reprogramming
ALKBH5	Direct conversion of m6A to A	Stabilize mRNAs of pluripotency factors and regulate RNA splicing and nuclear export to maintain ESC identity and support self-renewal
FTO/ALKBH9	Successive hydroxylation of 6mA to 6hmA, 6fA and A	Control mRNA stability and translation of stemness-related transcripts; influence proliferation and differentiation of neural and mesenchymal stem cells
LSD1/2 (KDM1A/B)	Demethylation of H3K4me_1/2_; H3K9me_1/2_	Coordinate enhancer decommissioning during differentiation by removing active histone marks; regulate bivalent promoter resolution to enable proper germ layer specification

*A* adenine, *ESC* embryonic stem cell, *FTO* a fat mass and obesity-associated protein, *HSC* hematopoietic stem cell, *m6A* N6-methyladenine, *5mC* 5-methylcytosine, *5hmC* 5-hydroxymethylcytosine, *5fC* 5-formylcytosine, *5caC* 5-carboxycytosine.

The JMJC domain serves as the catalytic core of JMJD histone demethylases and is responsible for removing histone methyl marks^90^. Structurally, the domain consists of a combination of α-helices and β-sheets, which form a pocket for the coordination of Fe²⁺ and α-KG, which are required for its catalytic function. The demethylation process occurs through a α-KG-dependent mechanism, in which oxygen is activated to oxidize the methyl group on the histone substrate. This reaction results in the production of formaldehyde and demethylated histones, which contribute to dynamic chromatin remodeling and gene expression regulation^91^.

The JMJN domain, which is located at the N-terminal region of certain JMJ enzymes, interacts with the JMJC domain to enhance enzymatic activity and provide structural stability. Although not universally present in all JHDM family members, it has an auxiliary role in supporting the catalytic machinery^92–94^.

JMJD histone demethylases are further distinguished by additional domains that define their substrate specificity and functional diversity. They facilitate interactions with histone modifications or DNA, enabling precise regulatory control. Some JMJDs have plant homeodomain fingers (for example, a plant homeodomain in KDM5A/RBP2 binds H3K4me3 to facilitate targeting that demethylase to its substrate). TUDOR domains present in KDM4/JMJD2 family members recognize methyl-lysines or methyl-arginines, which can help KDM4 enzymes localize to methylated chromatin regions^95^. Other modules such as ARID (AT-rich interaction domain) or zinc fingers can direct JMJDs to DNA elements or specific protein partners^94^. These modular domains ensure that each JMJ demethylase acts in a context-appropriate manner.

Each JMJ subfamily has distinct histone targets: for instance, KDM4 (JMJD2A-D) enzymes demethylate H3K9me3 (a heterochromatin mark) and H3K36me3 (an active mark), implying that they can either activate or repress transcription depending on context. KDM6 (UTX/JMJD3) specifically demethylates H3K27me3, the Polycomb-mediated repressive mark^96^. KDM5 (JARID1A-D) removes H3K4me2/3, generally acting as transcriptional repressors by erasing active marks^97^. The enzymatic breadth of the JMJDs allows dynamic regulation of both activating and repressive histone codes during cell state transitions.

##### Epigenetic role of the JMJD family in stemness

Various JMJD family enzymes have pivotal roles in biological processes, such as stem cell self-renewal, differentiation, and cell fate determination. Of these, JMJD3/KDM6B is particularly notable for its H3K27me3 demethylation activity, which contributes to the dynamic regulation of gene expression during differentiation and development^98^.

Although JMJD3/KDM6B is expressed during preimplantation, its knockdown paradoxically improves embryo quality, suggesting context-specific roles^99^. Moreover, pharmaceutical inhibition or targeted knockouts/knockdowns of JMJD3/KDM6B activate the DNA damage response, leading to cell death in differentiating cells, but not in undifferentiated mESCs^100^.

The absence of JMJD3/KDM6B has significant consequences for neurogenesis. In human neural progenitor cells, the lack of JMJD3/KDM6B results in the persistence of H3K27me3 in the promoter regions of neural and glial lineage genes, which causes transcriptional repression and disrupts neural development^101^. Human neural progenitor cells deficient in JMJD3/KDM6B fail to fully execute their developmental programs, which indicates the importance of dynamic H3K27 methylation during this process. Moreover, JMJD3/KDM6B expression is upregulated in neuroblasts and is essential for their differentiation into neurons. JMJD3/KDM6B deletion in subventricular zone NSCs in mouse during development and adulthood impairs neuronal differentiation, further highlighting its role in neurogenic processes^102^.

JMJD6 is essential for embryonic development and possesses histone arginine demethylation and lysyl hydroxylase activity^103,104^. BRD4 recruits JMJD6 to distal enhancers^105^. Notably, it can protect the productive transcriptional elongation of pluripotency genes in human and mouse ESCs^106^. JMJD6 supports pluripotency by sustaining glycolytic and pluripotency gene expression, and its overexpression enhances reprogramming efficiency. These results underscore the importance of JMJD6 in balancing stemness and differentiation, linking it to pluripotency and cellular identity^107^. In addition, JMJD6 promotes the maintenance of mHSC self-renewal by suppressing OXPHOS and other detrimental mechanisms, such as p53 stabilization^108^.

JMJD1C/KDM3C, a histone H3K9 demethylase, is highly expressed in hESCs. It directly regulates miR-302 expression, a microRNA involved in maintaining pluripotency and suppressing differentiation. JMJD1C/KDM3C knockdown in hESCs results in decreased bone morphogenic protein signaling and enhanced transforming growth factor-β signaling, which are pathways that maintain pluripotency and differentiation balance. Moreover, JMJD1C/KDM3C knockdown remains pluripotent, but exhibits a higher tendency toward neural differentiation^109^.

JMJD2/KDM4s are essential histone demethylases that regulate ESC self-renewal, pluripotency, and efficient iPSC generation. JMJD2B/KDM4B interacts with Nanog to maintain pluripotency, whereas JMJD2C/KDM4C collaborates with PRC2 for transcriptional repression. Both enzymes target distinct and overlapping chromatin regions. Their dynamic epigenetic regulation ensures the activation or repression of lineage-specific genes, which highlights their roles in maintaining mESC identity and facilitating cellular reprogramming^110^.

In preimplantation mouse embryos, JMJD2C/KDM4C is expressed in a stage-specific manner, peaking between the two-cell and eight-cell stages. It regulates chromatin modifications that are required for early embryonic development, including the activation of pluripotency genes. JMJD2C/KDM4C depletion in embryos results in significant downregulation of Nanog, Oct4, and Sox2, as well as cell proliferation genes, such as Myc and Klf4, causing developmental arrest before the blastocyst stage. These results highlight the indispensable role of JMJD2C/KDM4C as a histone demethylase that supports early embryonic progression and maintains pluripotency^111^.

#### RNA 6-methyladenine dioxygenases: ALKBH5 and FTO

##### Molecular properties

N6-methyladenosine (m6A) is the most prevalent internal modification of mRNAs, small nuclear RNAs, transfer RNAs, and ribosomal RNAs. It regulates various aspects of RNA metabolism and function^112–115^. This reversible modification has an important role in mRNA stability, splicing, translation, and decay, ultimately influencing gene expression and various cellular processes^116–118^. The addition of m6A is catalyzed by a methyltransferase complex primarily composed of METTL3, METTL14, and METTL16 (refs. ^119,120^). Conversely, its removal is catalyzed by specific demethylases, including ALKBH5 (AlkB homolog 5) and FTO (ALKBH9)^121,122^. Both ALKBH5 and FTO are iron (Fe²⁺)-dependent and α-KG-dependent dioxygenase enzymes belonging to the AlkB family (Fig. 4c).

ALKBH5 contains a conserved 2OG-Fe(II) dependent oxygenase domain that is common to the ALKBH family. It has a central role in demethylating m6A. This catalytic domain is characterized by a well-preserved DSBH domain that acts as a scaffold. The DSBH domain includes a HXD…H motif, which is essential for Fe²⁺ coordination, and an R…R motif, required for α-KG binding and substrate recognition. Moreover, this domain features a large loop structure, which physically obstructs the access of double-stranded DNA or RNA, thereby ensuring substrate specificity for single-stranded RNA^123^. In addition to the catalytic domain, ALKBH5 contains an alanine-rich sequence and a coiled-coil region within its N-terminal region, which may facilitate its nuclear localization^117^.

FTO was the first identified m6A demethylase, and its activity is not limited to mRNA but also extends to DNA and other methyladenosine-containing RNAs^124^. FTO, similar to other members of the ALKBH family, possesses the α-KG-Fe(II)-dependent oxygenase domain at its N-terminal, which includes the HXD…H and R…R motifs. In addition to these features, the N-terminal also contains nucleotide recognition lids and a loop structure, which contribute to substrate selectivity^125^. Uniquely, FTO harbors a non-homologous domain at its C terminus. This domain is thought to stabilize the structure of the N-terminal domain and may determine the substrate-binding specificity for molecules, such as transfer RNA and small nuclear RNA^126^.

To remove m6A, FTO sequentially oxidizes it to N6-hydroxymethyladenosine, followed by N6-formyladenosine. These intermediates are eventually hydrolyzed to adenine to complete the demethylation pathway^127^. This multistep process may enable cells to dynamically fine-tune m6A demethylation in response to physiological conditions. By contrast, ALKBH5 exhibits higher specificity for mRNA and is involved in the regulation of mRNA nuclear export, stability, and translation^121^. ALKBH5 directly removes the methyl group from m6A with formaldehyde release^128^, effectively converting it back to unmodified adenosine. The functional diversity of these enzymes highlights their importance in fine-tuning the epitranscriptomic landscape.

##### Epigenetic role of the ALKB family in stemness

m6A modifications in mRNAs are dynamically regulated during stem cell fate changes, and the balance of methylation/demethylation is critical. ALKBH5 and FTO ensure proper m6A levels on specific transcripts to fine-tune gene expression programs.

ALKBH5 is highly relevant in multiple stem cell systems. In normal and cancer stem cells, ALKBH5 sustains stem-like properties by demethylating transcripts involved in self-renewal. For instance, in human leukemia stem cells, ALKBH5 removes m6A from mRNAs such as SAV1, stabilizing them and promoting stem cell maintenance^129^. In human mesenchymal stem cells, ALKBH5 declines with age, and its overexpression rejuvenates aged cells by reducing m6A and restoring stemness^130^. Consistently, ALKBH5 depletion in hESCs biases differentiation toward mesoderm/cardiac lineages, as m6A accumulates on key mRNAs controlling those fate decisions^131^. ALKBH5 overexpression improves human mesenchymal stem cells engraftment and therapeutic effects in tissue injury models. Thus, ALKBH5 supports stem cell self-renewal and multilineage potential by preventing excessive m6A methylation that would otherwise mark transcripts for degradation or altered splicing.

ALKBH5 also has a protective role in certain stem cell niches; for example, it restrains activation of hepatic stellate cells in liver injury, thereby reducing fibrosis^132^. Taken together, these results highlight the central role of ALKBH5 in maintaining stemness across various biological systems. Its ability to enhance cancer stem cell self-renewal, while enabling rejuvenation and differentiation in normal stem cells, positions it as a target for regenerative medicine and cancer therapy.

FTO likewise influences stem cell biology. In NSCs, FTO modulates m6A on mRNAs that control neurogenesis; FTO knockout mice show impaired adult neurogenesis and behavioral defects^133^. In MSCs, FTO promotes osteogenic differentiation by demethylating and upregulating transcripts of pro-osteogenic factors (while inhibiting adipogenic pathways)^134^. In the context of malignancy, FTO is an oncogenic factor in certain leukemias and solid tumors: it demethylates m6A on transcripts such as *MYC* and *CEBPA* in AML, preserving their expression and supporting leukemia stem cell self-renewal^135^. FTO activity sustains therapy-resistant cancer stem cells in glioblastoma and pancreatic cancer^136,137^. FTO deficiency in mESCs reduces LINE1 RNA stability and shifts cells toward a two-cell-like transcriptional state^138^. FTO-deficient ESCs had decreased chromatin accessibility and lower expression of developmentally regulated transcripts. Pharmacological inhibition of FTO has shown promise in restraining cancer stem cells and could potentially push them toward differentiation or death^135^.

Both ALKBH5 and FTO are thus pivotal for maintaining the proper expression of genes required for stem cell maintenance versus differentiation, through their control of m6A marks. Mutations in these enzymes underscore their developmental importance: Alkbh5 knockout female mice have impaired fertility owing to defective RNA metabolism in germ cells^139^, and loss-of-function mutations in FTO in mice cause growth retardation, abnormalities in brain and cardiac development, and increased perinatal lethality^140–143^. These results highlight the important role of epitranscriptomic regulation in early development, reinforcing the importance of m6A dynamics in embryogenesis and organ differentiation.

#### FAD-dependent oxidoreductases: LSD1/2 (KDM1A/B)

##### Molecular properties

Both LSD1 and LSD2 contain an amine oxidase domain (AOD) that binds the FAD cofactor and carries out the redox-based demethylation reaction^144^. They oxidize the *N*-methyl group on lysine, forming an imine that hydrolyzes to formaldehyde and an unmethylated lysine (Fig. 4d). The flavin cofactor is reduced in the process and is regenerated by transferring electrons to O_2_, producing H_2_O_2_.

LSD1 and LSD2 contain an SWIRM domain (Swi3p, Rsc8p, and Moira) at the N terminus, which is involved in protein–protein interactions during the assembly of chromatin–protein complexes^145^. In addition to mediating protein interactions, the SWIRM domain interacts with the C-terminal AOD domain (AOD) to form a hydrophobic region, which contributes to the structure of the active site cavity^146^. The AOD domain serves as the catalytic core of the enzyme, facilitating the removal of methyl groups from the lysine residues on histones. In LSD1, the AOD domain contains an inserted tower domain, which forms a helical hairpin structure. This unique architecture divides it into two subdomains, each contributing to the catalytic activity of LSD1 by mediating substrate-binding and FAD binding, respectively^146^. Conversely, the AOD domain of LSD2 lacks a tower domain and exists as a single, continuous structure. Moreover, LSD2 features shorter solvent-exposed surface loops compared with LSD1, which results in differences in substrate selection^147^. Another distinctive structural feature of LSD2 is the CW-type zinc finger domain located at its N terminus. This domain interacts with the SWIRM domain to obstruct binding of the H3 tail. However, the zinc finger domain is connected to the SWIRM and AOD domains, forming a cohesive structure that maintains an active conformation, which enhances FAD binding, thereby promoting the demethylation activity^147^. The structural features of the active sites render them incompatible with trimethylated lysine residues. Furthermore, the catalytic mechanism, which is dependent on proton-dependent oxidation, cannot act on trimethylated lysine. Because of these structural and mechanistic constraints, LSD1 and LSD2 are limited to the demethylation of the monomethylated and dimethylated lysine residues^144^.

LSD1 demethylates transcription activation-associated H3K4me1/2 and repression-associated H3K9me1/2. Its intrinsic function is context-dependent and determined by the specific binding partners involved. For example, when LSD1 interacts with C-terminal binding protein (CTBP)^144,148^, COREST^149^, and the NURD complex^150^, it preferentially removes H3K4me1/2. Conversely, LSD1 is an activator by demethylating H3K9me1/2 with androgen receptor α and estrogen receptor α^151,152^. In addition, a neuron-specific isoform of LSD1, known as LSD1n, has FAD-dependent H4K20me1/2 demethylase activity, which is enhanced by its interaction with the COREST complex^153^. Unlike LSD1, LSD2 requires an extended region of the H3 tail for substrate recognition^154^. During this process, NAPC/GLYR1, a H3K36me3 reader, forms a tetramer to facilitate LSD2’s recognition of nucleosomes, thus enhancing its binding and processing efficiency^155^.

##### Epigenetic role of LSD1/2 in stemness

LSD1 is responsible for the demethylation of H3K4 and H3K9 and has a dual role in regulating gene expression through activation or repression^156^. In mouse ESC, LSD1 is present at active enhancer sites as part of the NURD complex, contributing to enhancer silencing. These enhancers are decommissioned during the transition from a pluripotent to a differentiated state. Loss of LSD1 activity inhibits full differentiation, because pluripotency-associated enhancers fail to deactivate, thus hindering the transition to a differentiated cellular state^157^.

Similarly, in human ESC, LSD1, in cooperation with Oct4 and Nanog, occupies developmental gene loci and facilitates the removal of H3K4me1/2. This creates a chromatin environment conducive to the addition of H3K27me3 by PRC2, highlighting the potential role of LSD1 in regulating bivalent promoters^158–160^. Moreover, LSD1 removes methyl groups from Dnmt1, thus contributing to its stability. Taken together, LSD1 serves as a coordinator between histone methylation and DNA methylation.

Consistent with its significance, LSD1 deficiency disrupts early embryogenesis, particularly during gastrulation, highlighting its essential role in development^161^. LSD1 is also indispensable for NSC proliferation. Its regulation of NSC proliferation involves recruitment to target gene promoters through its interaction with TLX, a nuclear receptor and regulator of NSC activity, thereby ensuring a role for LSD1 in neural development^162^.

Moreover, LSD1 activity affects the differentiation of various cell types, such as HSCs, insulin-producing cells, adipogenic cells, and myogenic cells.

Unlike LSD1, LSD2 is predominantly expressed in differentiated cells and has a role in lineage determination. In beige adipocyte progenitors derived from white adipose tissue, LSD2 knockdown upregulates genes associated with myogenesis, rather than adipogenesis. This suggests that LSD2 suppresses alternative lineage pathways during adipocyte differentiation^163^.

The role of LSD2 in various cancer types has been extensively studied, particularly its association with cancer stem cell enrichment. In basal-like breast tumors, LSD2 expression is higher compared with other breast cancer subtypes or normal breast tissue. This increased expression is correlated with rapid proliferation, but is inversely associated with cellular motility, including migration and invasion^164^. In gastric cancers, LSD2 is overexpressed because of miR-3196 inhibition by ADPGK-AS1 (ADPGK antisense RNA 1), which contributes to cancer progression by promoting cellular proliferation and migration^165^. In addition, LSD2 was identified as a dysregulated epigenetic modifier in hepatocellular carcinoma tissues^166^.

LSD2 also functions as an E3 ubiquitin ligase, independent of its demethylase activity. It directly ubiquitylates O-GlcNAc transferase to promote its proteasome-dependent degradation. The E3 ligase activity of LSD2 inhibits the growth of A549 lung cancer cells. These results indicate that LSD2 regulates distinct sets of genes through its dual functions, with specific pathways influenced by its demethylase activity and others through its E3 ubiquitin ligase activity^163^.

#### Brief summary

Oxidoreductases emerge not merely as metabolic supporters but as direct executors of epigenetic change. Their chromatin-associated activities, especially demethylation reactions, translate metabolic conditions into structural and functional chromatin outcomes with high fidelity. Because these enzymes require metabolites such as α-KG, FAD, and oxygen, their activity is inherently sensitive to metabolic fluctuations. Thus, this section highlights oxidoreductases as molecular bridges that convert metabolic signals into immediate chromatin remodeling decisions. This provides mechanistic insight into how the energy contour is physically written onto the genome, reinforcing the conceptual framework. To visualize how these enzymatic activities are integrated within specific cellular contexts, we summarize the distinct metabolic and epigenetic characteristics of key stem cell states in Table 3. This comparison illustrates how the metabolic landscape of each cell state shapes and sustains its specific epigenetic program.

**Table 3:** Comparison of epigenetic and metabolic characteristics across stem cell states.

Feature	Naive pluripotent stem cells	Primed pluripotent stem cells	Adult stem cells	Cancer stem cells
Cell state	Ground state	Poised state	Quiescence	Malignant proliferation and resistance
Primary energy metabolism	Bivalent metabolism OXPHOS (oxidative phosphorylation) + glycolysis + fatty acid oxidation	Glycolytic dependence Aerobic glycolysis	Hypometabolism Fatty acid oxidation and OXPHOS	Metabolic plasticity switching between glycolysis and fatty acid oxi dation
Mitochondria	Active, elongated	Inactive, fragmented	Functional (suppressed reactive oxygen species (ROS))	Dysfunctional or biosynthetic
Acetyl-CoA source	Glucose + fatty acids (active pyruvate dehydrogenase (PDH) and fatty acid oxidation (FAO))	Glucose (ACLY pathway, minimal FAO)	Fatty acids (FOXO1–CPT1A axis)	Variable (context-dependent)
α-Ketoglutarate (α-KG) supply	Isocitrate dehydrogenase (IDH) activity (mito and cyto)	Glutaminolysis	Glutamine and glucose	IDH mutation (2-hydroxyglutarate (2-HG)) or glutamine
α-KG/succinate ratio	High (promotes demethylation)	Low (inhibits demethylation, succinate accumulation)	Regulated (maintains quiescence)	Dysregulated (inhibited by 2-HG)
DNA methylation level	Hypomethylation (active TET, ten-eleven translocation methylcytosine dioxygenase (TET) enzymes)	Hypermethylation (suppressed TET enzymes)	Context-specific (lineage repression)	Aberrant hypermethylation (TET loss of function)
Histone state	Global hyperacetylation (high H3K9ac, H3K27ac)	Bivalent domains High histone lactylation (H3K18-lac)	Hypoacetylation (low H4K16ac)	Aberrant modifications High lactylation, mixed repressive marks
Key metabolic enzymes	PDH (active), IDH2 (active), *N*-methyltransferase (NNMT)	Pyruvate dehydrogenase kinase (PDK) (PDH inhibition), lactate dehydrogenase (LDH) (active), indoleamine 2,3-dioxygenase 1 (IDO1)	CPT1A (FAO control), aldehyde dehydrogenase (ALDH) (high)	Mutant IDH, FTO, ALDH (high)
Key regulators	STAT3, TFE3, MOF	HIF1α, c-MYC, aryl hydrocarbon receptor	FOXO1, mTOR (low)	c-MYC, HIF1α, mutant IDH
Metabolo-epigenetic consequence	Open chromatin, pluripotency maintenance	Poised state, glycolytic gene activation	Lineage repression, stem cell pool maintenance	Tumor suppressor silencing, dedifferentiation and stemn ess

## Non-enzymatic oxidoreductases

### Dehydrogenases as scaffold proteins for epigenetic complexes

#### An NADH-dependent dehydrogenase CTBP

##### Molecular properties

**Fig. 5:**
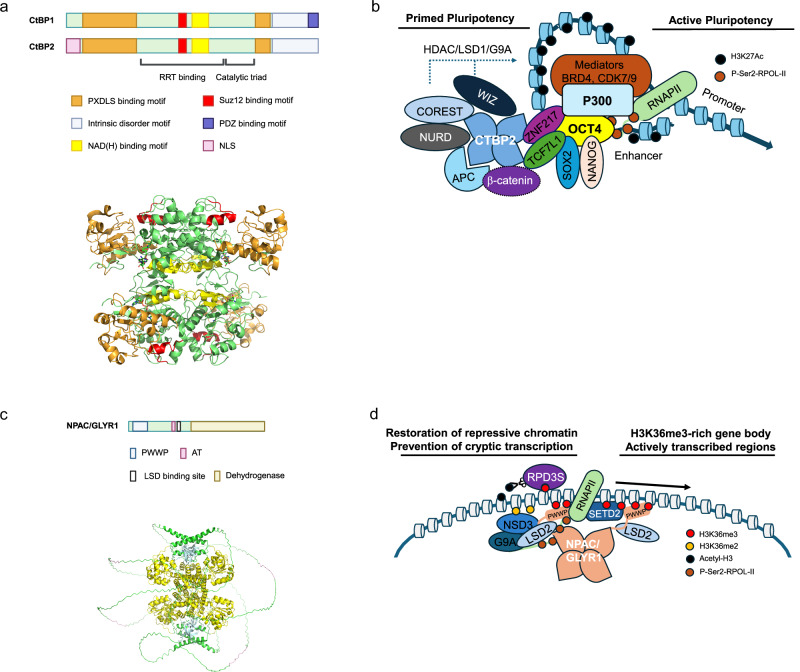
Schematic model of CTBP and NPAC/GLYR1 as epigenetic regulators in ESCs. **a** Primary structure of CTBP isoforms and 3D structure of tetrameric CTBP2 (PDB ID: 6WKW). Each of the binding motifs is marked with matched colors. The dehydrogenase domain (green) makes up the tetrameric form. **b** Schematic model of CTBP2 in the enhancer region of the pluripotent mouse embryonic stem cell (mESC) genes. CTBP2 primes the pluripotent mESC genes by linking zinc finger transcriptional repressors with various transcriptional co-repressors in the pluripotent mESC genes. **c** Primary and 3D structures of tetrameric NPAC/GLYR1 predicted by AlphaFold-3. The dehydrogenase domain (yellow) makes up the tetrameric form. **d** Schematic model of NPAC/GLYR1 as a transcriptional elongation factor. NPAC/GLY1 associates with active RNA polymerase II and its PWWP domain recognizes H3K36me3, which is catalyzed by SETD2 during transcriptional elongation. LSD2 associates with the dodecapeptide linker (214–225) in NPAC/GLYR1. NPAC/GLYR1-LSD2 further recruited epigenetic regulators, possibly due to the restoration of repressive chromatin states following RNA polymerase II passage near the transcription start site regions. HDAC, histone deacetylase; LSD, lysine-specific demethylase.

CTBP was initially identified through its interaction with the C terminus of the adenoviral E1A protein, thereby repressing its transforming activity^167^. Two CTBP isoforms, CTBP1 and CTBP2, show high homology in their primary and quaternary structures. CTBP typically consists of three domains: a substrate-binding domain at the N terminus, a dehydrogenase domain in the middle, and an intrinsically disordered region at the C terminus. A unique characteristic of CTBP is its ability to recognize the pentapeptide PXDLS motif in various interacting partners. In addition to the PXDLS motif, the SUZ12-binding motif (amino acids 160–173 in CTBP2) is located in the middle of the dehydrogenase domain, close to the substrate-binding domain in the quaternary structure^168^ (Fig. 5a).

CTBP1 was initially reported to form dimers in the presence of NAD(H)^169^. Further studies confirmed that NAD(H)-bound CTBP predominantly exists as dimers^170–172^; however, additional evidence indicated that NAD(H) promotes the assembly of CTBP into tetramers^173–177^ (Fig. 5a). The tetrameric nature of CTBP allows for extensive interactions with multiple proteins simultaneously. This acts as a scaffold to gather DNA-binding transcription factors with epigenetic regulators on chromatin. With respect to epigenetic regulation, CTBP functions as a transcriptional repressor by associating with nucleosome remodeling and the deacetylase (NURD) complex^148^, class II HDACs (HDAC4/5/7/9)^178^, and COREST (co-repressor of REST), which includes HDAC1/2 and LSD1, and removes acetyl groups and H3K4 monomethylation/dimethylation from nucleosomes, leading to transcriptional repression^179^ (Fig. 5b). CTBP associates with p300/CBP and inhibits their HAT activity^180^ and recruits the histone methyltransferases GLP and G9A (EHMT1/2) through WIZ to repress target genes in mESCs^181^. WIZ interacts with cohesin and binds active enhancers and promoters^182^, consistent with CTBP2 occupancy at these regions in mESCs^183^.

##### The epigenetic role of CTBP2 in stemness

CTBP2 in mESCs was discovered as 1 of 10 common OCT4-associated proteins from four independent studies using mass spectrometry^184–187^. Of the CTBP1 and CTBP2 isoforms, only CTBP2 knockout mice showed severe developmental defects, dying by E10.5 from problems with extra-embryonic development, whereas the CTBP1 mutants were viable, although smaller^188^. In support of this, CTBP2 knockdown delayed differentiation upon LIF withdrawal and altered mesodermal differentiation^189^. Furthermore, large-scale small interfering RNA screens identified CTBP2 as a gene regulating ESC commitment^190^.

CTBP interacts with a wide variety of transcription factors, most notably, zinc finger transcription factors (ZFTs). These ZFTs often regulate genes involved in stemness, such as the maintenance of stem cell pluripotency, development, and tumorigenesis. Recent studies have shown that CTBP2 occupies H3K27ac-enrichded enhancer regions of active ESC genes and associate with HDAC complexes (NURD, COREST-LSD1) to modulate gene expression^183,191^. Interestingly, CTBP2 coexists with the core transcription factors Oct4/Sox2/Nanog (OSN) at the enhancer regions of active ESC genes. CTBP2, along with ZNF217, primes active ESC gene regions by recruiting NURD–LSD1 complexes to repress gene expression during differentiation. Similar functions are likely performed by ZNF516 and HIC2. Knockout of either ZNF217 or ZNF516 disrupts ESC differentiation^191^.

In mESCs, CTBP2 knockdown stabilizes β-catenin, enhancing its association with OSN and delaying exit from pluripotency^192^. CTBP2 co-occupies with pluripotency gene enhancers and TCF3 (TCF7L1), a major TCF family member in early embryonic development^192,193^. TCF3 depletion inhibits differentiation by inducing Nanog expression^194^. Similarly, CTBP2 knockout in mESCs does not affect pluripotency maintenance, but alters differentiation timing, possibly by allowing β-catenin greater access to OCT4-binding sites^168,191^. CTBP2 also modulates β-catenin through α-catenin and adherens junctions, contributing to integrated control of signaling and chromatin regulation^192,195^. By contrast, CTBP knockdown in primed hESCs reduces self-renewal and pluripotent markers^196^. This suggests that the role of CTBP in APC/β-catenin differs between naive and primed pluripotency. Notably, stabilized β-catenin in naive mouse and human ESCs promotes self-renewal by enhancing OCT4 activity independently of TCF3^197,198^, whereas in primed hESCs and mEpiSCs, stabilized nuclear β-catenin induces differentiation^199,200^. This suggests that WNT/β-catenin promotes efficient self-renewal and inhibits the transition of naive to primed states.

CTBP2 associates with PRC2 components. Knockdown of CTBP2 prevents the formation of H3K27me3 regions around active ESC genes following LIF withdrawal^183^. This indicates that CTBP2 likely recruits PRC2 to active ESC gene regions to repress their expression during differentiation. Recently, CHK2 was shown to phosphorylate serine at position 164 (Ser164) in CTBP2, which resulted in the disassembly of the tetramer into monomers following DNA damage^168^. Phosphorylated monomeric p-CTBP2(S164) loses its binding affinity to HDAC1, LSD1, and ZNF217. Instead, it preferentially binds to PRC2 through direct interaction with SUZ12, an essential component of PRC2. This suggests that CTBP2 prevents gene silencing from PRC2-mediated repression of active ESC genes during DNA damage.

#### NADH-dependent dehydrogenase NPAC/GLYR1

##### Molecular properties

NPAC/GLYR1 was initially identified as nuclear protein 60 (ref. ^201^). It consists of multiple domains, including a PWWP domain (residues 1–105) followed by an A-T hook (residues 168–180) at the N terminus, a linker segment (residues 214–225) in the middle, and a dehydrogenase (NAD-binding) domain (residues 261–553) at the C terminus^155^ (Fig. 5c). NPAC/GLYR1 was independently discovered as a GLYR1, which belongs to the oxidoreductase family in plants. NPAC/GLYR1 contains an NAD^+^-binding domain at the C terminus, which is homologous to the β-hydroxyacid dehydrogenase superfamily^155^.

The dehydrogenase domain of NPAC/GLYR1 forms a tetramer as a four-leaf clover-shaped dimer of dimers. The Rossmann fold in NPAC/GLYR1 (residues 262–437) binds tightly to NADH or NADPH; however, a lysine residue in the catalysis region, which is strongly preserved in β-hydroxyacid dehydrogenases, is replaced by a methionine residue in NPAC/GLYR1. The replacement of a methionine residue at position 437 to lysine (M437K) in NPAC/GLYR1 resulted in a monomer by analytical size-exclusion chromatography^155^ and triggered a structural conformation that led to the disassembly of dimer formation^202^. These results demonstrate that the catalytically inert dehydrogenase domain in NPAC/GLYR1 functions as a scaffold for the proper assembly of epigenetic partners (Fig. 5d).

The function of NPAC/GLYR1 was initially characterized by its ability to bind to the trimethylated histone H3-K36 (H3K36me3) as an epigenetic factor (Fig. 5d). A deletion analysis revealed that the PWWP domain of NPAC/GLYR1 is necessary for H3K36me3 binding^203^, and NPAC/GLYR1 localizes to the H3K36me3 loci in chromatin. The PWWP domains of other nuclear proteins (BRPF1, DNMT3a, PSIP1/LEDGF, and HRP3) have been shown to bind to H3K36me3 (refs. ^204–207^). Interestingly, the PWWP-NPAC/GLYR1 domain interacts with naked DNA with high affinity, enabling NPAC/GLYR1 to bind in vitro to nucleosomes regardless of whether H3K36 is methylated. An A-T hook in conjunction with the PWWP domain participates in nucleosome binding^155^. NPAC/GLYR1 was independently identified as a nucleosome-destabilizing factor. Its PWWP domain interacts with nucleosomes near the dyad, destabilizing them in an ATP-dependent manner and facilitating transcription by Pol II through nucleosomes^208^.

LSD2 directly binds the dodecapeptide linker (residues 214–225) within the disordered region of NPAC/GLYR1 (ref. ^209^). It associates with Ser2-phosphorylated and Ser5-phosphorylated RNA polymerase II, P-TEFb, and CDK9, all of which are involved in transcriptional regulation. LSD2 binding correlates with high levels of H3K36me3 in actively transcribed genes in HeLa cells^210,211^. Similarly, NPAC/GLYR1 overlaps with H3K36me3-rich gene bodies in both HeLa cells and mESCs^203,208,211^, suggesting that the NPAC/GLYR1–LSD2 complex functions as a transcription elongation factor. This is consistent with H3K36me3 being a hallmark of active transcription^212,213^. However, as many genes with moderate-to-high H3K36me3 levels are not enriched in NPAC/GLYR1, H3K36me3 alone is likely insufficient for its chromatin recruitment^208^. Nonetheless, more detailed studies are necessary to understand how epigenetic enzymes collaborate with NPAC/GLYR1 to distribute histone codes in actively transcribed genes during transcriptional elongation.

##### The epigenetic role of NPAC/GLYR1 in stemness

In mESCs, NPAC/GLYR1 suppresses the expression of pluripotency genes such as Nanog and Rif1. Its depletion disrupts transcriptional elongation of these genes, impairing ESC pluripotency maintenance^211^.

Alternative splicing was shown to regulate ESC pluripotency, reprogramming, and lineage differentiation^214,215^. In NPAC/GLYR1 knockout cells, embryoid body formation is reduced compared with that in wild-type cells, which suggests an impaired capacity for proper differentiation^216^. NPAC/GLYR1 associates with the splicing factor SRSF1, facilitating the assembly of the early spliceosome for alternative splicing^217^. NPAC/GLYR1 knockout results in aberrant alternative splicing patterns, highlighting the role of NPAC/GLYR1 in alternative splicing during ESC pluripotency and differentiation^216^.

The mechanism by which alternative splicing links histone modifications to the stem cell fate decision is unclear^218^. PSIP1/LEDGF contributes to the regulation of alternative splicing by linking the splicing factor SRSF1 to chromatin by recognizing H3K36me3 through its PWWP domain^219^. PSIP1/LEDGF-SRSF1 switches PBX1a to PBX1b to determine hESC fate^218^. NPAC/GLYR1 also recognizes H3K36me3 through its PWWP domain; thus, NPAC/GLYR1 may similarly contribute to ESC fate. Future studies will be necessary to understand the molecular mechanism through which NPAC/GLYR1 regulates alternative splicing during transcriptional elongation.

#### Brief summary

This section broadens the concept of metabolic–epigenetic integration beyond catalytic reactions, demonstrating that oxidoreductases also act as structural sensors of the cellular redox state. Through NAD(H)-dependent oligomerization and protein–protein interactions, scaffold-type dehydrogenases regulate the composition and activity of chromatin-modifying complexes in ways that are independent of metabolic flux itself. Their function illustrates that metabolic state does more than supply substrates or limit enzymatic reactions. It can directly reorganize the chromatin regulatory machinery.

These scaffold roles complete a conceptual framework in which metabolic processes generate essential cofactors, catalytic enzymes use those cofactors to modify chromatin, and structural interactions further modulate chromatin-associated complexes. Together, these layers provide a comprehensive explanation for how metabolic status can restructure the epigenome across multiple regulatory dimensions.

## Discussion and perspectives

Stem cells with identical genetic information must establish precise spatiotemporal gene expression control mechanisms according to their developmental stage, internal and external conditions, and interactions with surrounding cells. This regulation is necessary for maintaining cellular identity and is primarily governed by epigenetic regulation, which enables the reversible control of gene expression to ensure that cells adapt appropriately to their environment, in which metabolism is the most important factor. Cells, even microorganisms, adjust their metabolism in response to environmental cues, such as nutrient availability, oxygen tension, and their relationship with neighboring cells, to promote survival.

It remains unclear whether metabolic states primarily drive epigenetic changes, or whether epigenetic mechanisms are the dominant regulators influencing cellular metabolism. It has been hypothesized that metabolism was evolutionarily prioritized, given that microorganisms lacking advanced epigenetic systems maintain central carbon metabolic pathways that closely resemble those of mammals. In this context, it is plausible that core metabolic pathways predated epigenetic regulation and that epigenetic mechanisms are developed by incorporating metabolic enzymes and metabolites. In mammals, this long-standing metabolic–epigenetic relationship appears to have become further refined to support complex developmental and environmental adaptation. This evolutionary perspective provides a conceptual framework for understanding why specific metabolic enzymes have become integral components of epigenetic regulation, setting the stage for the following discussion of oxidoreductase-mediated mechanisms.

Oxidoreductases have important roles in epigenetic regulation in three areas. First, they are responsible for the production of epigenetic metabolites. Dehydrogenases participate in central carbon metabolism and are directly involved in producing acetyl-CoA and α-KG. They are also involved in SAM synthesis and the one-carbon cycle. These metabolites are primarily considered essential for energy production and as building blocks for biomolecules. Therefore, it is important to consider why such important metabolites have evolved to be used as epigenetic sources. It is hypothesized that in a certain evolutionary era, surplus energy in the form of metabolites was attached to DNA, providing benefits that were essential for survival. For example, surplus acetyl-CoA may function by marking histones in the chromatin, thereby genetically encoding the metabolic activity level in the cell. Hyperacetylation in histones reflects a high metabolic state in PSCs, which leads to gene activation for the maintenance of pluripotency. As oxidoreductases are primarily responsible for energy flow in metabolic pathways, these metabolites may reflect the potential energy state within the cell to form an energy contour that is ultimately expressed as an epigenetic contour on chromatin and determines the final epigenetic landscape.

Second, oxidoreductases catalyze the removal of the methyl group attached to histone/DNA/RNA. Generally, epigenetic regulation operates with an enzymatic pair with the transferase-hydrolase system. Writer proteins are primarily transferases, including HAT, DNMT, poly-ADP ribosyltransferases, and O-GlcNAc transferase, whereas the erasers, such as HDAC, SIRTs, poly-ADP-ribose glycohydrolase, and O-GlcNAcase, are hydrolases that remove both acetyl and O-GlcNAc groups (Fig. 1b). Hydrolases can remove moieties attached to lysine and nucleotides using water.

Oxidoreductases catalyze the hydroxylation of the methyl group attached to histone/DNA/RNA in an O_2_/α-KG-dependent manner. These epigenetic oxidoreductases resemble the bacterial Alkb DNA repair enzyme^220^. Nine ALKBHs (1–9) exist for repairing various alkylated nucleotides in mammals. Of these, ALKBH5 and ALBH9/FTO act as epigenetic enzymes by removing the methyl group from m6A in RNA. Other isoforms function as DNA damage repair enzymes^221^. In addition, TET enzymes remove the methyl group by successive hydroxylation of 5mC on DNA; however, 5fC and 5caC are finally removed by the base excision repair enzyme, thymine-DNA glycosylase^71,72^ (Fig. 4a). JMJD histone demethylases also remove the methyl group from a lysine residue using similar mechanisms. Repairing histone modifications is not necessarily as urgent as repairing DNA and RNA. Thus, these epigenetic oxidoreductases may be bifurcated from the repair enzymes. In fact, nucleotide methylation in prokaryotes has evolved by protecting their own DNA, because the methyl group has several advantages, such as being small, but thermodynamically stable, to avoid foreign attack^222^. Conversely, the removal of the methyl group from lysine and nucleotides requires high energy, and molecular oxygen paired with α-KG acts as an electron acceptor for this reaction. Thus, demethylation of the methyl group in lysine or DNA occurs in the local area of chromatin, unlike the global distribution of histone acetylation. In fact, naive PSCs maintain low levels of methylated DNA; however, TET demethylase activity is not essential for the maintenance of pluripotency. Instead, TET isozymes are required for proper differentiation by generating 5hmC at local regions^85^.

Third, some NADH-dependent dehydrogenases serve as scaffold proteins in epigenetic complexes. Interestingly, they contain a Rossmann fold that binds NAD(H), but lack enzymatic activity toward specific substrates. For NPAC/GLYR1, an important lysine residue in the active site of the β-HDH family is replaced by methionine, and it reverts to lysine, resulting in a transition from a tetramer to a monomer. CTBP has known substrates, such as MTOB, but there is no definitive evidence that its enzymatic activity directly contributes to the epigenetic regulatory functions of CTBP. Thus, why did these NADH-dependent dehydrogenases evolve to have a role in epigenetic complexes? In many cases, NADH-dependent dehydrogenases are known to undergo monomer–dimer–tetramer transitions depending on NAD(H) concentration. This suggests that they may induce changes in the protein quaternary structure, which reflects intracellular NADH levels. In fact, both CTBP and NPAC/GLYR1 recruit epigenetic proteins in their tetrameric form to carry out their functions in mESC pluripotency.

Oxidoreductases have very important roles in metabolic pathways aligned with the characteristics of stem cells and are directly involved in determining the epigenetic landscape specific to stem cell traits. However, a deeper understanding is still required regarding their precisely coordinated roles during the stemness (or pluripotency) transition. Therefore, achieving a precise understanding of this will reveal how metabolic changes affect the epigenetic state and, in turn, whether they are involved in determining pluripotency.

## Conclusion

Oxidoreductases serve as central mediators linking cellular metabolism to epigenetic regulation in stem cells. By producing key metabolites, catalyzing demethylation reactions, and scaffolding chromatin-modifying complexes, they dynamically shape the epigenetic landscape that governs stemness and differentiation. The integration of metabolic flux with chromatin regulation underscores the evolutionary coupling of energy metabolism and gene control. Future studies aimed at dissecting the context-specific roles of individual oxidoreductases will not only deepen our understanding of stem cell biology but also open new therapeutic avenues in regenerative medicine and cancer.
